# Population Pharmacokinetic Model of AST-001, L-Isomer of Serine, Combining Endogenous Production and Exogenous Administration in Healthy Subjects

**DOI:** 10.3389/fphar.2022.891227

**Published:** 2022-06-24

**Authors:** Soyoung Lee, Su-Kyeong Hwang, Hee-Sook Nam, Jung-Sook Cho, Jae-Yong Chung

**Affiliations:** ^1^ Department of Clinical Pharmacology and Therapeutics, Seoul National University College of Medicine, Seoul, South Korea; ^2^ Kidney Research Institute, Seoul National University Medical Research Center, Seoul, South Korea; ^3^ Department of Pediatrics, School of Medicine, Kyungpook National University, Daegu, South Korea; ^4^ Astrogen Inc., Daegu, South Korea; ^5^ Department of Clinical Pharmacology and Therapeutics, Seoul National University Bundang Hospital, Seongnam, South Korea

**Keywords:** population pharmacokinetics, L-serine, simulations, model-informed dose selection, autism

## Abstract

AST-001 is an L-isomer of serine that has protective effects on neurological disorders. This study aimed to establish a population pharmacokinetic (PK) model of AST-001 in healthy Korean to further propose a fixed-dose regimen in pediatrics. The model was constructed using 648 plasma concentrations from 24 healthy subjects, including baseline endogenous levels during 24 h and concentrations after a single dose of 10, 20, and 30 g of AST-001. For the simulation, an empirical allometric power model was applied to the apparent clearance and volume of distribution with body weight. The PK characteristics of AST-001 after oral administration were well described by a two-compartment model with zero-order absorption and linear elimination. The endogenous production of AST-001 was well explained by continuous zero-order production at a rate of 0.287 g/h. The simulation results suggested that 2 g, 4 g, 7 g, 10 g, and 14 g twice-daily regimens for the respective groups of 10–14 kg, 15–24 kg, 25–37 kg, 38–51 kg, 52–60 kg were adequate to achieve sufficient exposure to AST-001. The current population PK model well described both observed endogenous production and exogenous administration of AST-001 in healthy subjects. Using the allometric scaling approach, we suggested an optimal fixed-dose regimen with five weight ranges in pediatrics for the upcoming phase 2 trial.

## Introduction

Autism spectrum disorder (ASD) is a neurodevelopmental disorder characterized by cognitive deficit and motor impairment (Lord et al., 2020). Current therapy is mainly focused on reducing symptoms to improve patient’s learning ability and relieve aggressive behavior. Two psychotropic drugs, risperidone and aripiprazole, are the mainstays of ASD treatment, approved by the Food and Drug Administration (FDA) for use in irritability associated with autistic disorder (LeClerc and Easley, 2015). However, both medications can only manage symptoms of severe irritability and aggression and cannot treat the core signs and symptoms of ASD (Anagnostou, 2018). In addition, long-term treatment with risperidone and aripiprazole is required to control these symptoms, which increases the risk of dyskinesias and can induce weight gain and sedation, leading to discontinuation of the treatment (Troost et al., 2005; McPheeters et al., 2011; Lord et al., 2020).

AST-001 is under development by Astrogen Inc. (Daegu, South Korea) to treat children with ASD based on their body weight. The main active pharmaceutical ingredient is L-serine, one of the isomers of non-essential amino acids, which can be synthesized *in vivo* and supplied through diet. AST-001 plays a critical source in the biosynthesis of nucleotides, glycine, cysteine, phosphatidylserine, and sphingolipids, which are essential for maintaining the normal central nervous system (de Koning et al., 2003; Mattaini et al., 2016). It also showed protective effects against oxidative damage in mitochondria and neuronal cells (de Koning et al., 2003; Gantner et al., 2019). Sufficient intake through food is required because *de novo* synthesis cannot meet the cellular demands for its use (de Koning et al., 2003). In particular, low activity of 3-phosphoglycerate dehydrogenase (3-PHGDH) can cause synthetic disorder of AST-001, leading to abnormal brain development and function (Sayano et al., 2013; Mattos et al., 2015; El-Hattab et al., 2016). Thus, researchers have been interested in applying amino acids for the treatment of neurodegenerative diseases (Garofalo et al., 2011; Levine et al., 2017).

We expect that AST-001 will improve symptoms of cognitive function, social skills, and behaviors in patients with ASD. As it is an amino acid, long-term treatment is generally safer than other atypical antipsychotic drugs. Currently, there are limited pharmacokinetic (PK) data to characterize the endogenous and exogenous amino acid profiles in humans (Bier, 2003; Hasegawa et al., 2019; Miller et al., 2020). In this study, we aimed to establish a population PK model to elucidate both endogenous production and exogenous oral administration of AST-001 in healthy Korean male subjects. Based on the model, we simulated various multiple dosing scenarios in the pediatric population using allometric scaling to achieve sufficient systemic exposure to AST-001. We proposed a fixed-dose regimen based on weight range for the upcoming phase 2 trial in pediatric patients.

## Materials and Methods

### Data

Data from a phase I parallel study in healthy subjects were separately used for model building and additional validation. The PK model was constructed using data of 24 subjects treated with a single dose of 10, 20, or 30 g AST-001 and their serial baseline endogenous levels before treatment. For additional evaluation, the same clinical data from seven subjects after a single and twice-daily multiple doses of 15 g AST-001 were used to validate the model performance. Serial blood samples were obtained at 0, 10, 20, 30, 45 min, 1, 1.5, 2, 3, 4, 6, 9, 12, 24 h before administering AST-001 to assess baseline endogenous levels on day -1. Subjects were sampled up to 24 h at the same time points of baseline assessment on day 1 after receiving a single dose of AST-001 (10, 15, 20, or 30 g) suspended in 200 ml of water around 9 a.m. Blood samples were obtained at 0, 10, 20, 30, 45 min, 1, 1.5, 2, 3, 4, 6, 9, 12, 24, 48 h after 7-days multiple administrations of AST-001 (15 g twice daily). Because AST-001 is an amino acid, intake of high-protein meals was prohibited during the study. The trial was conducted in accordance with the Declaration of Helsinki and approved by the Ministry of Food and Drug Safety and the Institutional Review Board of Seoul National University Bundang Hospital (B-2002-597-001). The study was prospectively registered in the Korean Open Clinical Trial Registry (Clinical Research Information Service, KCT0005915). All subjects provided informed consent prior to participation in the study.

### Determination of Plasma AST-001

The plasma concentrations of AST-001 were determined by validated liquid chromatography (LC-30AD, AB Sciex, United States) coupled to mass spectrometry (Triple Quad 4500, AB Sciex, United States). The test compound was separated using the Porpshell 120 EC-C18 column (3.0 mm × 50 mm; particle size, 2.7 μm) and detected via electrospray ionization with positive ion mode in multiple-reaction-monitoring. The plasma sample for AST-001 was protein precipitated and AST-001-d3 was used as an internal standard (IS). To separate AST-001, the composition of the mobile phase was (A) 0.2% formic acid in water and (B) acetonitrile, and flow rate was 0.2 ml/min. The m/z transition for AST-001 and IS was 106.0 → 60.1 and 109.1 → 63.1, respectively. The calibration curve was calculated through linear regression using the ratio of the peak area of the AST-001 to that of the IS. The calibration curve was linear over the range of 4–800 μg/ml. The range of accuracy and precision of assay was 95.5–102.8%, and 1.6–4.3%, respectively.

### Pharmacokinetic Model Development

The population PK model of AST-001 was developed using nonlinear mixed-effects modeling using NONMEM (version 7.4.0, ICON Development Solutions, Ellicott City, United States) with Pirana (version 2.9.9, Certara, Princeton, United States) interface (Keizer et al., 2013; Beal et al., 2016). PK parameters and their variabilities were estimated using the first-order conditional estimation method with the interaction option. R software (R Core Team, 2020) was used to handle data, perform statistical analysis, and generate figures (Team, 2020). Individual PK profiles were explored graphically to determine whether there were any outliers in the dataset. Although two subjects showed relatively higher endogenous baseline levels and fluctuation (Supplementary Figure S1), they were all included in the analysis. Methods for processing censored data were not considered because all of the measured concentrations were above the lower limit of quantification (4 μg/ml).

Several potential structural models of orally administered AST-001 were investigated, including one or two compartmental models with first- or zero-order absorption applying linear or non-linear (Michaelis-Menten) elimination. Because the overall baseline level did not show a significant sign of circadian rhythm (Supplementary Figure S1), the production of endogenous AST-001 was assumed to be a zero-order process in the steady-state. The model was considered significantly improved, if the objective function value (OFV) was decreased more than 3.84 (*p* < 0.05, χ^2^ distribution with 1 degree of freedom). The exponential error model was used to assess the inter-individual variability of PK parameters. The covariance between the random effects were also evaluated. Eta shrinkage was calculated and considered acceptable if the values are less than 30%. Inter-occasion variability was not considered during the model development, because the baseline levels and PK sampling after dose were obtained in the consecutive days during the admission. Various residual error models were explored, including additive, proportional, and both combined.

Body weights were screened as a potential covariate to affect the PK of AST-001. The power model was applied to test body weight effects, and the conventional forward selection (*p* < 0.05, decreased in OFV >3.84) and backward elimination (*p* < 0.01, increased in OFV >6.63) methods were used to determine the retention of the covariate. If the estimated allometric scaling coefficients were not feasible with the empirical values, body weights were not included in the final model regardless of statistical significance.

### Pharmacokinetic Model Evaluation

Model selection and evaluation were based on both numerical and graphical diagnostics, including precision of estimates, comparison of the OFV, goodness-of-fit plots, visual predictive check, and normalized prediction distribution error (NPDE). The model adequacy was demonstrated graphically by comparing the observed plasma concentration to that of the simulated 5%, median, and 95% prediction in the 10, 20, and 30 g dose groups. Non-compartmental analysis results were compared to determine the parameter appropriateness. The predictive performance of the model was assessed using additional validation datasets by graphically comparing the observed concentration to the simulated time-concentration profile of AST-001 after single and twice-daily multiple doses of 15 g. The robustness of the final model was confirmed by bootstrap analysis, where the dataset was resampled 1,000 times. The estimated median values and 95% confidence intervals of each parameter were compared with the estimates from the original dataset. The model was determined to be stable if the estimated values were not significantly different and confidence intervals are reasonably narrow.

### Model-Based Simulation for Adult and Pediatric Populations

The simulation was conducted with the final PK model to predict the time-concentration profiles of AST-001 after single and 7-days multiple twice-daily administrations of 10, 15, 20, and 30 g doses, and baseline endogenous levels in adults. Because the average half-life of AST-001 ranges from 7 to 14 h, multiple doses over 7 days are considered sufficient to achieve a steady state. Two hundred subjects were simulated in each dose group scenario, and PK parameters were calculated by non-compartmental analysis using the NonCompart R package (version 0.4.7) (Kim et al., 2018). To assess the systemic exposure of externally administered AST-001, the time-matched baseline was adjusted, and the negative value after baseline adjustment was replaced with 0. Following the individual’s baseline, adjusted PK parameters were calculated: maximum plasma concentration (C_max_), C_max_ at steady state (C_max,ss_), area under the curve (AUC) from 0 to 12 h after single dose administration (AUC_12h_), AUC over the dosing interval (AUC_τ_), and trough concentration (C_trough_).

To explore the fixed-dose regimen according to the weight range of pediatric patients, we applied an empirical allometric power model for simulation (Equation 1) (Liu et al., 2017).

$$Parameter_{pediatric}=Parameter_{adult}\text{∗}{\left(\frac{Body\;Weight_{pediatric}}{70}\right)}^{\theta }$$ (1)

Because the estimated allometric scaling parameters were not plausible with the empirical values, we fixed the exponential scale of 0.75 to apparent clearance and inter-compartmental clearance and a scale of 1 to the central and peripheral volume of distribution. The time-concentration profile of AST-001 after 7-days twice-daily doses of 1–15 g was simulated with a weight range of 10–60 kg. Each weight and fixed-dose scenario was simulated for 100 subjects. We proposed a dosing regimen of AST-001 by weight range, based on the descriptive statistics for AUC_τ_, in which it achieves similar systemic exposure to AST-001 in a steady state after 15 g twice-daily (BID) dose in healthy adults.

## Results

### Study Population of Model Dataset

Model construction data included a total of 648 plasma AST-001 concentration values from 24 healthy male volunteers in phase I clinical trial. Among them, 336 observations were baseline endogenous concentrations, while 312 observations were concentration values after AST-001 administration. Model validation set included 308 plasma AST-001 concentrations from the same trial, where 98 were baseline, 91 were concentrations after a single dose of AST-001 15 g, and 119 were concentrations after multiple 15 g BID doses. The median (min-max) values of demographic characteristics in the datasets were as follows: age, 25 (19–51) years; height, 174.2 (161.4–182.6) cm; weight, 72.5 (54.5–86.9) kg; BMI, 23.8 (19.5–28.9) kg/m^2^.

### Population Pharmacokinetic Model

**FIGURE 1:**
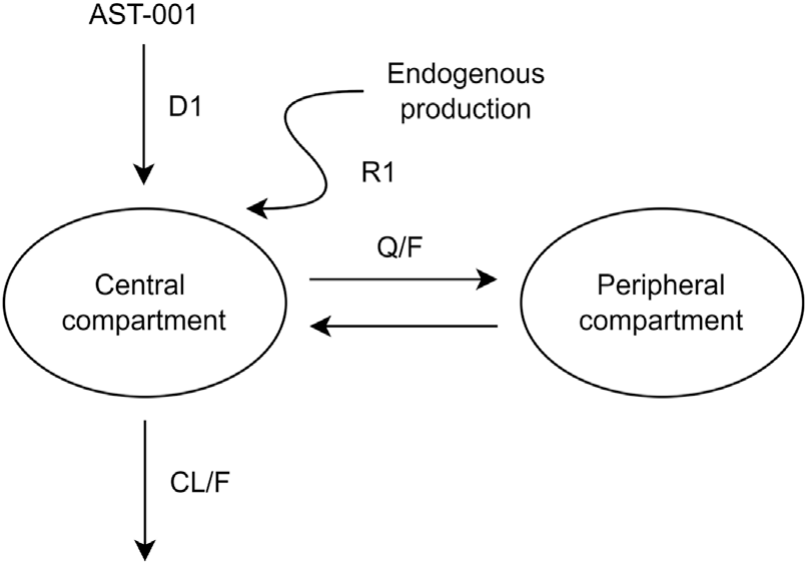
A pharmacokinetic model describing orally administered AST-001 and endogenous production. CL/F, apparent clearance; Q/F, apparent inter-compartmental clearance between the central and peripheral compartments; D1, duration of zero-order absorption; R1, rate of endogenous production of AST-001.

The PK model of orally administered AST-001 was appropriately described using a two-compartment model, zero-order absorption, and linear elimination with proportional residual variability (Figure 1). Endogenous AST-001 was well explained by continuous zero-order production, in which the rate of endogenous production (R1) was estimated to be 0.287 g/h (Figure 1; Table 1). The model estimated the duration of zero-order absorption (D1) approximately 1.26 h, which well described the rapid absorption of orally administered AST-001. The final estimates of apparent clearance (CL/F), apparent central volume of distribution (V1/F), apparent peripheral volume of distribution (V2/F), and apparent intercompartmental clearance (Q/F) were consistent with the non-compartmental analysis results after 15 g BID doses of AST-001, where CL/F and Vd/F were 20.6 L/h and 292.5 L, respectively. The relative standard error of population PK parameter estimates ranged from 5.8 to 11.6%, indicating good precision (Table 1). The inter-individual variability (IIV) of PK parameters (V1/F, CL/F, D1, R1) were included assuming log-normal distributions, and a variance-covariance block between V1/F and CL/F was also estimated. The PK parameters showed low shrinkage of IIV, implying less uncertainty, where eta shrinkage of V1/F, CL/F, D1, and R1 was 8.3, 5.1, 3.1, and 13%, respectively. Weight had a positive relationship with CL/F and V1/F; however, including those covariates did not significantly improve the model (Supplementary Table S1).

**TABLE 1:** Parameter estimates and bootstrap results of the final pharmacokinetic model of AST-001.

Parameter	Estimate	RSE (%)	Bootstrap median (95% CI)^a^
**Population parameters**			
V1/F (L)	68.4	6.7	67.8 (59.0–75.5)
CL/F (L/h)	22.9	5.8	22.8 (20.9–24.9)
V2/F (L)	196	11.6	198.8 (165.5–242.7)
Q/F (L/h)	16.4	7.3	16.6 (14.7–18.8)
D1 (h)	1.26	7.6	1.27 (1.13–1.54)
R1 (g/h)	0.287	6.2	0.284 (0.259–0.311)
**Inter-individual** **variability** ^b^			
V1/F	29.0	13.1	27.9 (20.6–33.8)
CL/F	22.2	23.9	21.1 (12.0–29.2)
D1	38.0	13.6	37.6 (26.7–47.8)
R1	18.0	23.6	17.4 (9.9–24.0)
Covariance between V1/F and CL/F^c^	0.0483	41.8	0.0444 (0.0173–0.0758)
**Residual Error**			
Proportional residual error^d^	0.183	7.5	0.182 (0.160–0.204)

RSE, relative standard error; CI, confidence interval; V1/F, apparent volume of distribution in the central compartment; CL/F, apparent clearance; V2/F, apparent volume of distribution in the peripheral compartment; Q/F, apparent inter-compartmental clearance between the central and peripheral compartments; D1, duration of zero-order absorption; R1, rate of endogenous production of AST-001.

a Results of bootstrap resampling for 1000 replicates.

b Inter-individual variability is presented as coefficient of variation (%).

c Untransformed estimates of covariance between V1/F and CL/F.

d Proportional residual error is presented as coefficient of variation.

**FIGURE 2:**
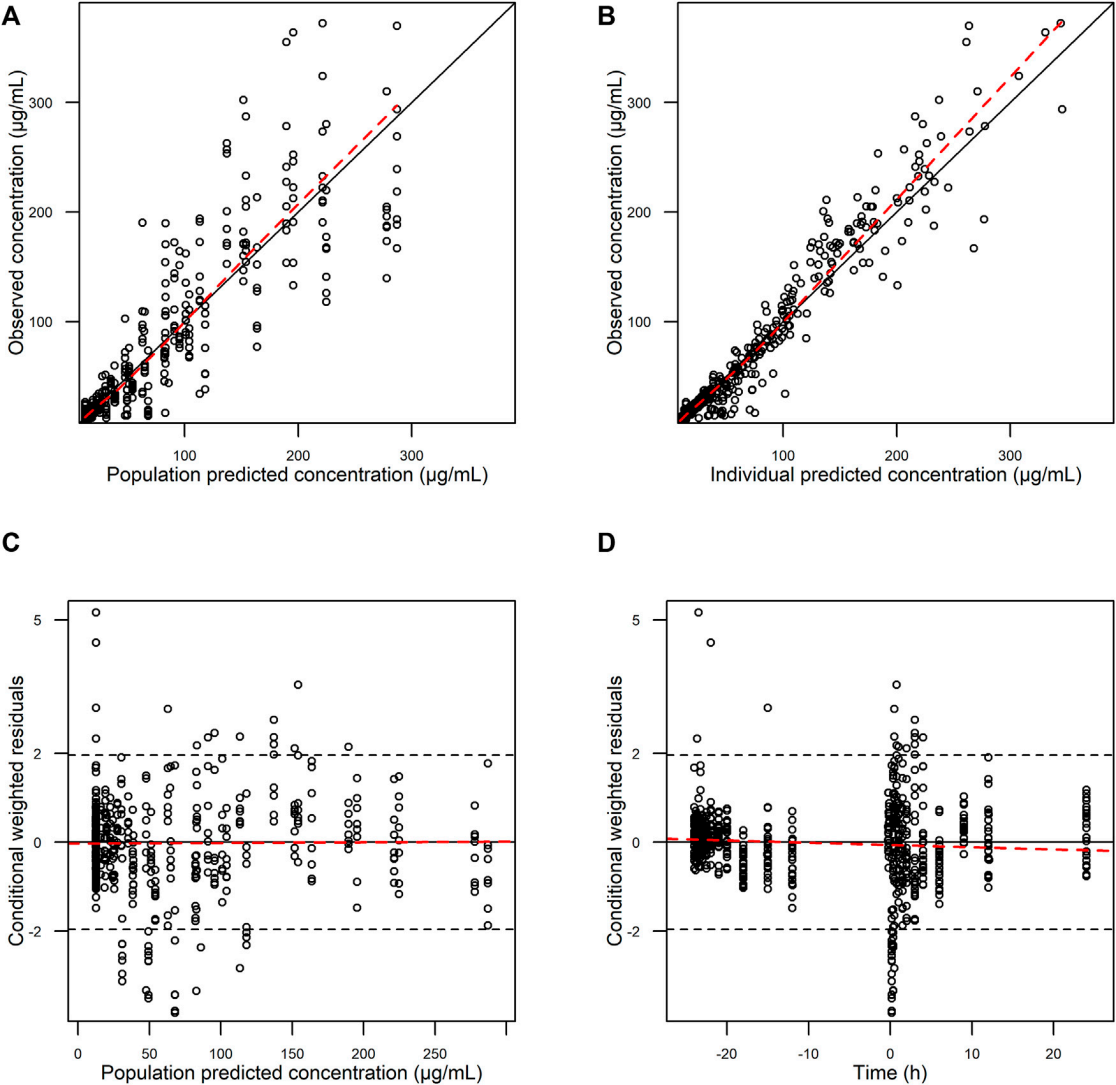
Basic goodness-of-fit plots of final AST-001 PK model. **(A)** observations versus population predictions; **(B)** observations versus individual predictions; **(C)** conditional weighted residuals versus population predictions; **(D)** conditional weighted residuals versus time.

**FIGURE 3:**
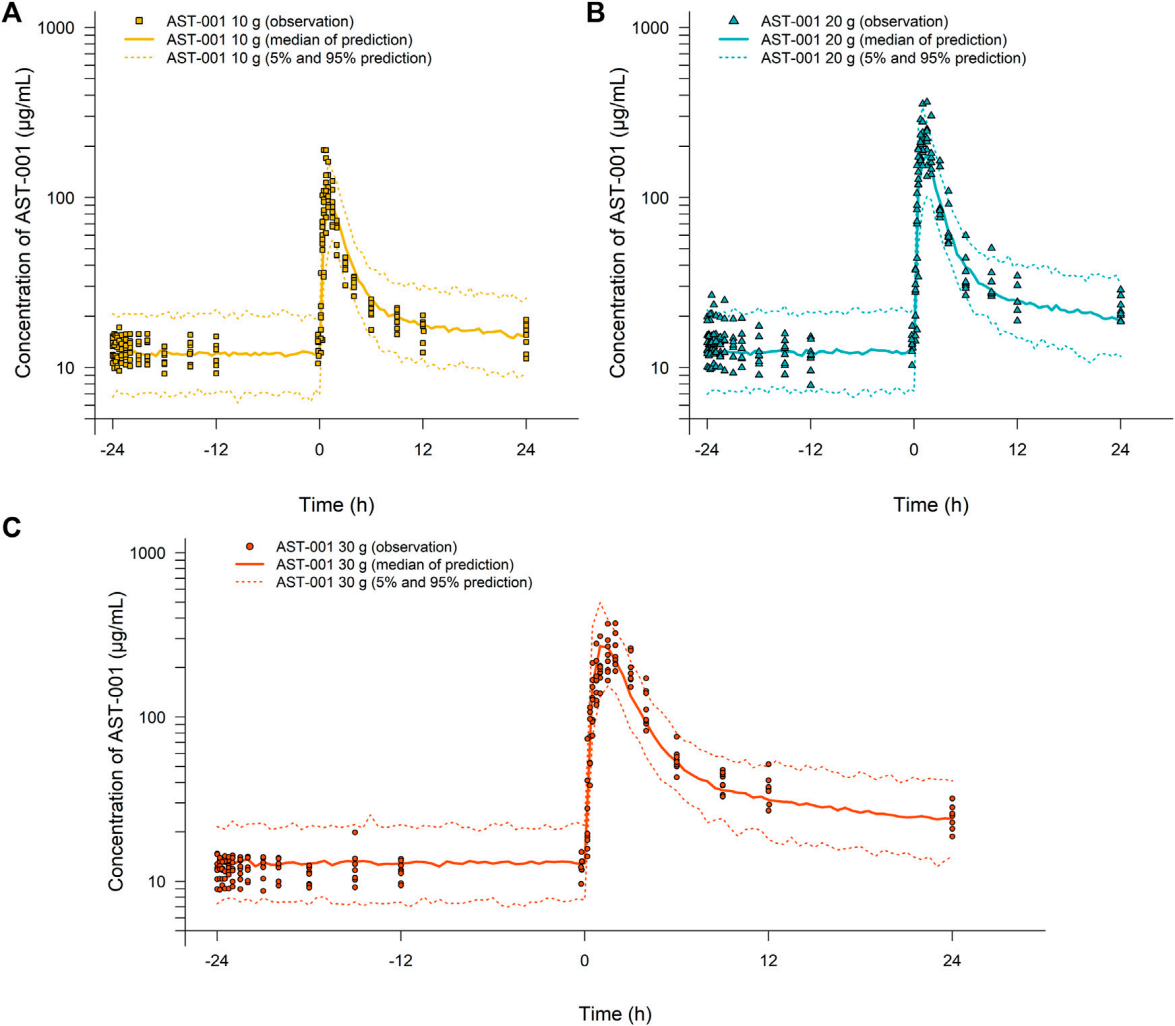
Simulation results for the time-concentration profile of AST-001 before and after a single administration of **(A)** 10 g, **(B)** 20 g, or **(C)** 30 g of AST-001 overlaid with individual observations in healthy subjects. Line and dotted line indicate median, 5 and 95% prediction intervals, respectively.

The goodness-of-fit plots showed an adequate model structure for predicting the AST-001 concentration obtained from healthy males (Figure 2). Two subjects who showed a high variation of baseline AST-001 level resulted in high conditional weighted residuals (Supplementary Figure S1, Supplementary Figure S2). The individual AST-001 concentration fell within the 95% prediction interval in each dose group scenario, supporting the model predictability (Figure 3). Likewise, the prediction-corrected visual predictive check plots stratified by dose group also showed that the observed AST-001 concentrations were within the 95% prediction intervals of the simulation (Supplementary Figure S3). The NPDE distributions also supported the adequate model performance (Supplementary Figure S4). The medians and 95% confidence intervals (CIs) of PK parameters generated by bootstrap were similar to the final PK parameter estimates, indicating model stability (Table 1). Overall, the model was robust and adequate, with good precision, to characterize the PK properties of AST-001.

### Simulation of Single and Multiple Administration of AST-001 in Adults

Model performance was also confirmed by evaluating the ratio between the simulated and observed median baseline-adjusted AUC_12h_. The ratio ranged from 0.93 to 1.13 across the single dosing scenario, implying the simulated baseline-adjusted AUC_12h_ was similar to that of observed AUC_12h_. The individual AST-001 concentrations of the additional validation sets fell within the 95% prediction interval for a single 15 g dose of AST-001 (Supplementary Figure S5). In the multiple dosing scenario, the PK model slightly under-predicted the C_max,ss_ after twice-daily administration of 15 g of AST-001 (Supplementary Figure S5). The observed median baseline-adjusted C_max,ss_ and AUC_τ_ were 225.5 μg/ml and 730.0 h*μg/mL, respectively, while the corresponding simulated median values were 179.2 μg/ml and 638.7 h*μg/mL.

**FIGURE 4:**
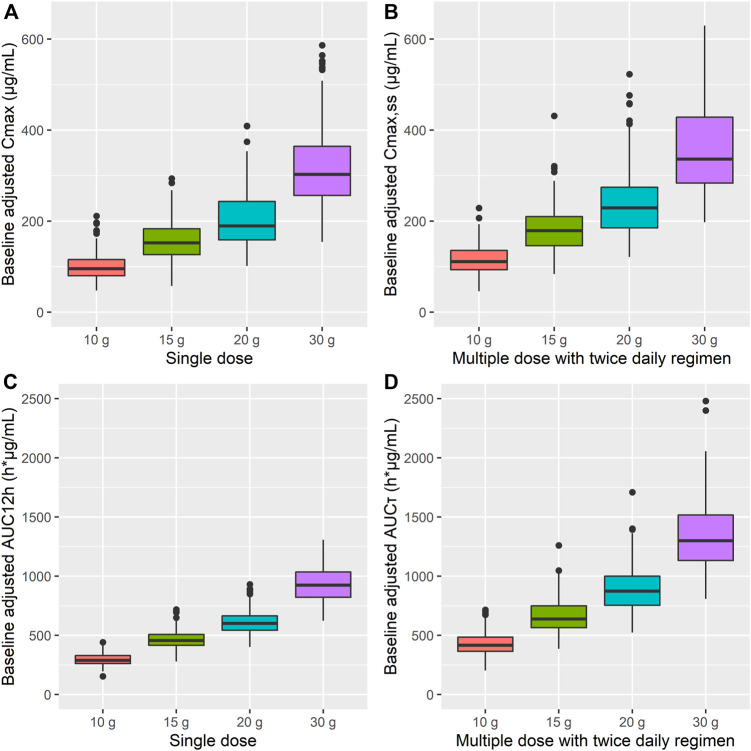
Simulation results for baseline-adjusted **(A)** C_max_, **(B)** C_max,ss_, **(C)** AUC_12h_, and **(D)** AUC_τ_ after single and 7-days twice-daily multiple administration of 10, 15, 20, and 30 g doses of AST-001 in healthy subjects. The dots are outliers that exceed the 25^th^ and 75^th^ quartile range. C_max_, maximum plasma concentration; C_max,ss_, C_max_ at steady state; AUC_12h_, area under the curve (AUC) from 0 to 12 h; AUC_τ_, AUC over the dosing interval.

Based on the simulation results, C_max_ and AUC_12h_ were increased by approximately 20 and 50%, respectively, after multiple administrations of AST-001 compared to single-dose administration (Figure 4, Supplementary Table S2). The simulated trough level mimicked the observed data after 15 g of twice-daily administration, regardless of baseline adjustment (Supplementary Figure S6). The simulated median C_trough_ of AST-001 were 26.0, 34.0, 42.0, and 56.0 μg/ml after a twice-daily dose of 10, 15, 20, and 30 g, respectively, while the corresponding baseline-adjusted C_trough_ were 13.5, 21.4, 29.1, and 43.2 μg/ml (Supplementary Figure S6).

### Simulation of Multiple Administration of AST-001 in Pediatric Population

**FIGURE 5:**
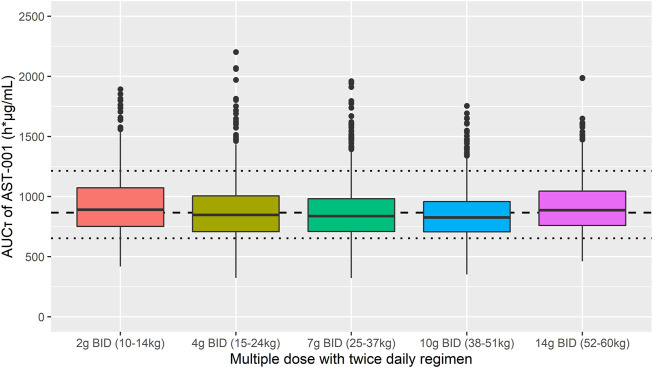
Simulation results of AUC_τ_ after 7-days twice-daily multiple administration of 2 g, 4 g, 7 g, 10 g, and 14 g in 10–14 kg, 15–24 kg, 25–37 kg, 38–51 kg, and 52–60 kg weight ranges, respectively. Short dashed line represents the median observed value after 7-days twice daily multiple doses of 15 g AST-001 in healthy subjects. Dotted line represents the corresponding minimum and maximum observed values. The dots are outliers in the simulation that exceed the 25^th^ and 75^th^ quartile range.

We assessed the target attainment percentage for each dose and weight scenario and categorized the weight range into five groups, where target attainment was achieved for more than 70% of patients. The target exposure was established as 653–1214 h*μg/mL based on the observed minimum and maximum values of AUC_τ_ in adults after a 15 g BID regimen. The simulated 2 g, 4 g, 7 g, 10 g, and 14 g BID regimens in the 10–14 kg, 15–24 kg, 25–37 kg, 38–51 kg, and 52–60 kg groups, respectively, showed that 74.0, 74.9, 77.2, 78.1, and 82.7% of simulated patients fell within the target exposure to AST-001 (Figure 5). Descriptive statistics of C_max,ss_ and AUC_τ_ for each simulated group also showed similar exposure between weight-ranged fixed-dose group (Supplementary Table S3).

## Discussion

In this study, a two-compartment PK model with zero-order absorption and linear elimination with continuous zero-order production of endogenous AST-001 was developed and qualified in a dataset of 24 healthy subjects. The final model of AST-001 was robust and adequate with good precision based on the goodness-of-fit plots, visual predictive checks, and bootstrap results. In addition, we compared the estimated median values of baseline-adjusted PK parameters after a single dose of 10–30 g and multiple doses of 15 g BID with those of observed values. As a result, simulated PK parameters were similar to those observed in clinical trials; therefore, our model well described the endogenous and exogenous profile of AST-001 in healthy subjects.

Currently, there are limited *in vivo* data to characterize endogenous amino acid levels and PK profiles after oral administration. To the best of our knowledge, three population PK models of glutamate, arginine, and serine have been reported in healthy subjects (Brussee et al., 2016; Wang et al., 2018; Bosley et al., 2021). Wang et al. described arginine PK with a first-order elimination two-compartment model, while glutamate PK was proposed with a nonlinear elimination one-compartment model (Wang et al., 2018). Because both PK models did not include endogenous production of amino acids, the terminal phase where the externally administered dose was cleared and the circulating endogenous amino acids remained was not well explained.

The PK model of L-serine, the same active ingredient as AST-001, was reported by Bosley et al., where the final model was one-compartment, first-order absorption, and elimination (Bosley et al., 2021). Similar to our study, they applied a constant parameter to explain the endogenous biosynthesis of L-serine. This model estimated an endogenous production rate of 440 mg/h, slightly higher than the rate of our model (287 mg/h), while we were more consistent with 252 mg/h estimated from Neis’s model (Neis et al., 2017). Bosley’s model included a relatively small number of subjects’ PK data after a “cocktail” of nicotinamide riboside, L-carnitine, N-acetyl cysteine, and serine with few baseline samples. Since our study included intensive 24 h baseline and PK sampling after various doses with more subject data, we were able to assess IIV of endogenous production (18%), and dose-linearity. In addition, our population PK analysis showed that L-serine was distributed to the peripheral compartment (196 L) more than in the central compartment (68.4 L) after oral administration. Compared to our study, Bosley described it as a one-compartment model, where the central compartment (218 L) accounted for the distribution. The elimination rate constant was similar to Bosley’s model estimate of 0.31 h^−1^.

In another population PK model of arginine in malaria patients, the natural time course of amino acid recovery was explained by a second-order polynomial model (Yeo et al., 2008; Brussee et al., 2016). This model was applied because the infectious process led to an increased turnover of amino acids, and as it subsided, the amino acid levels returned to baseline values (Yeo et al., 2008). Because our study was performed in healthy subjects with no infectious condition, the turnover model or other empirical polynomial models were not considered. Although food intake can affect the circulating levels of amino acids (Bier, 2003), the baseline levels of AST-001 showed a relatively constant value over 24 h. As *de novo* synthesis (6 g/day) is 2.4 fold higher than the average daily intake from food (2.53 g/day), diet may have a low impact on baseline AST-001 levels (Cox and Metcalf, 2017; Neis et al., 2017). In addition, we restricted the diet to minimize the possible influence of meals during the study. We forbid high protein intake and provided low-protein meals containing 25 g of protein and fat of less than 35% (total 800 kcal). Similarly, glutamine and alanine levels did not show time-dependent variations in healthy subjects (Klassen et al., 2000). Thus, continuous zero-order production with inter-individual variability well explained the endogenous production of AST-001.

In our model, we described the endogenous production and exogenous administration of AST-001 as independent processes. There is a report that serine concentration itself may regulate its synthesis by the negative feedback of phosphoserine phosphatase (Fell and Snell, 1988). This theory is controversial because several later studies have shown that serine levels did not affect the efflux of serine from the kidney (Lowry et al., 1987; Brosnan and Hall, 1989; Brundin and Wahren, 1994), which is the predominant source of endogenous production of serine (Pitts and MacLeod, 1972; Kalhan and Hanson, 2012). As our model well predicted the systemic exposure to L-serine after multiple administration of AST-001, the repeated dosing may have a negligible impact on the rate of endogenous L-serine production.

Previous studies have investigated serine doses for the treatment of 3-PHGDH deficiency and amyotrophic lateral sclerosis (ALS). Generally higher dose regimen of 500–600 mg/kg/day was required to benefit seizure control in patients with 3-PHGDH deficiency (de Koning et al., 1998; de Koning et al., 2000; de Koning et al., 2004). Likewise, a high dose of 15 g BID showed efficacy and tolerability in ALS patients (Levine et al., 2017; Bradley et al., 2018). We assumed that the efficacious dose in ASD would be similar to ALS patients, as its main pharmacological effect is neuroprotection (Cox and Metcalf, 2017; Dunlop and Carney, 2021). Because target concentration range is not established, we set the efficacy target with the observed exposure range of 653–1214 h*μg/mL after the 15 g BID regimen in adults. For future clinical study, we simulated 1–15 g BID doses with a weight range of 10–60 kg for pediatrics. As AST-001 showed relatively stable plasma L-serine concentration between age of 2–18 years (Lepage et al., 1997), we applied empirical allometry for body weight on PK parameters of externally administered AST-001. Practically, a fixed-dose regimen has more advantages in clinical settings than weight-based doses. Our simulation results showed that five weight ranges with a fixed-dose regimen would be sufficient to achieve observed AUC_τ_ in healthy adults after a 15 g BID regimen. Moreover, 74.0, 74.9, 77.2, 78.1, and 82.7% values of simulated 2 g, 4 g, 7 g, 10 g, and 14 g BID regimens for the respective group of 10–14 kg, 15–24 kg, 25–37 kg, 38–51 kg, and 52–60 kg fell within target exposures (Figure 5). Based on the simulation, a fixed-dose regimen may be applied in the upcoming phase 2 trial in the pediatric population.

A limitation of our study was that the population PK model was developed and validated with limited weight range and age; therefore, extrapolation of the model will require caution. Because we only included healthy subject data with limited weight range, it was impossible to identify clinically relevant covariates affecting the PK and baseline endogenous levels of AST-001. Further phase 2 study data involving pediatric patients with ASD could explain covariates affecting inter-individual PK variability and improve the clinical application of this model.

## Conclusion

The population PK model for AST-001 well described the continuous production of endogenous AST-001 and the disposition of exogenous AST-001 by a 2-compartment model with zero-order absorption and first-order elimination. The current model can be useful in determining dose regimens for populations with various weight ranges, such as children. Further model development using pediatric patient data will improve the clinical implications.

## Data Availability

The original contributions presented in the study are included in the article/Supplementary Material, further inquiries can be directed to the corresponding author.
