# Supplementary material for: Population Pharmacokinetic Model of AST-001, L-Isomer of Serine, Combining Endogenous Production and Exogenous Administration in Healthy Subjects
Source: Front Pharmacol. 2022 Jun 24;13:891227. doi: 10.3389/fphar.2022.891227 (PMC9263096; doi:10.3389/fphar.2022.891227)
Supplement: Supplementary file 1 [file DataSheet1.pdf]

Supplementary Material

1 Supplementary Figures and Tables

1.1 Supplementary Figures

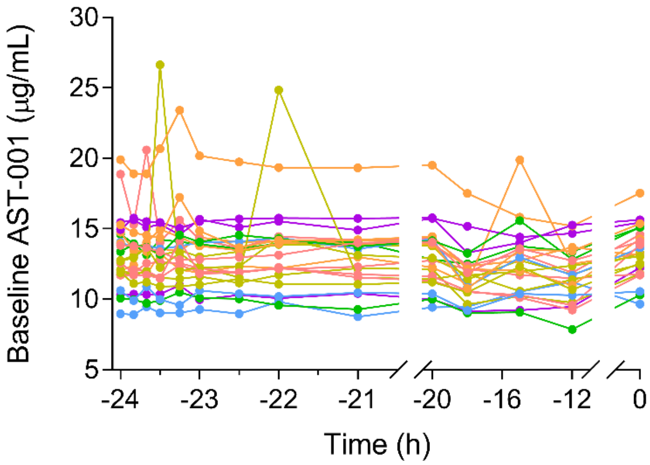

Supplementary Figure 1. Individual plasma concentration-time profile of AST-001 before oral administration of AST-001 in healthy subjects.

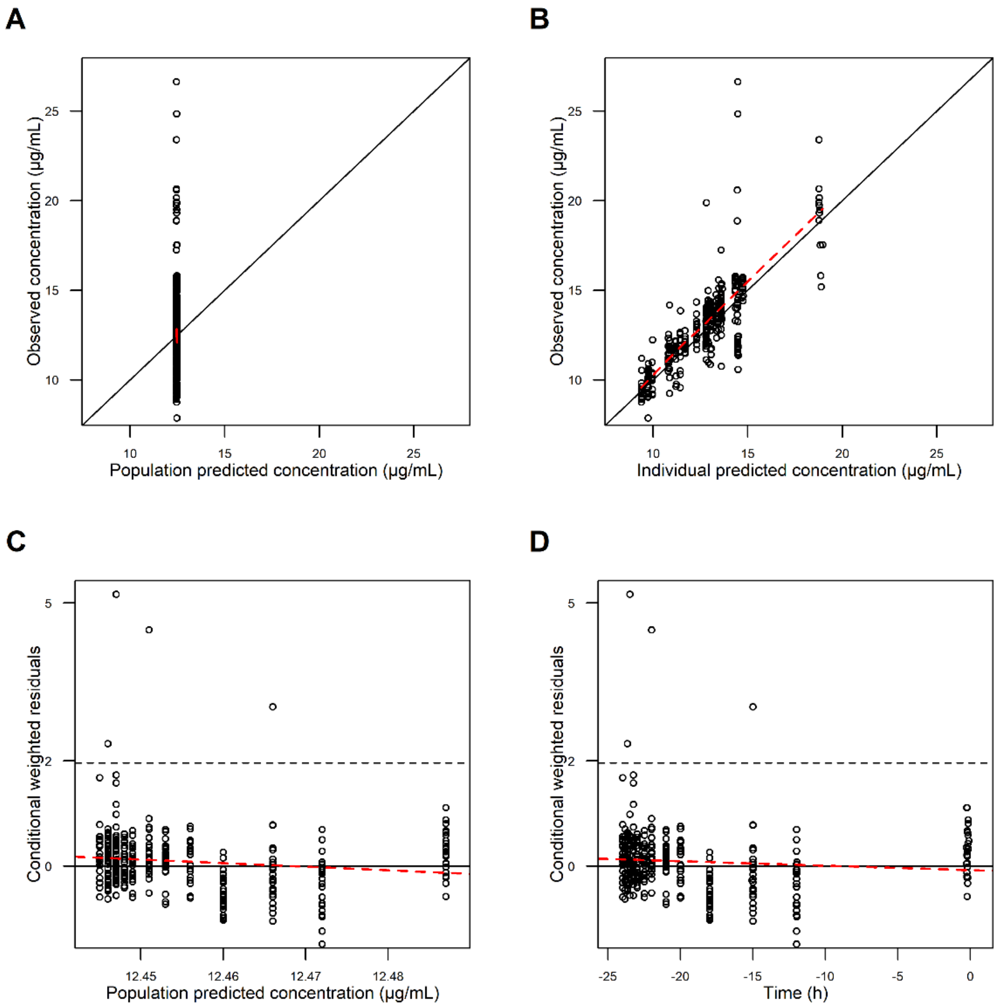

Supplementary Figure 2. Basic goodness-of-fit plots of final AST-001 PK model using baseline endogenous AST-001 data. (A) observations versus population predictions; (B) observations versus individual predictions; (C) conditional weighted residuals versus population predictions; (D) conditional weighted residuals versus time.

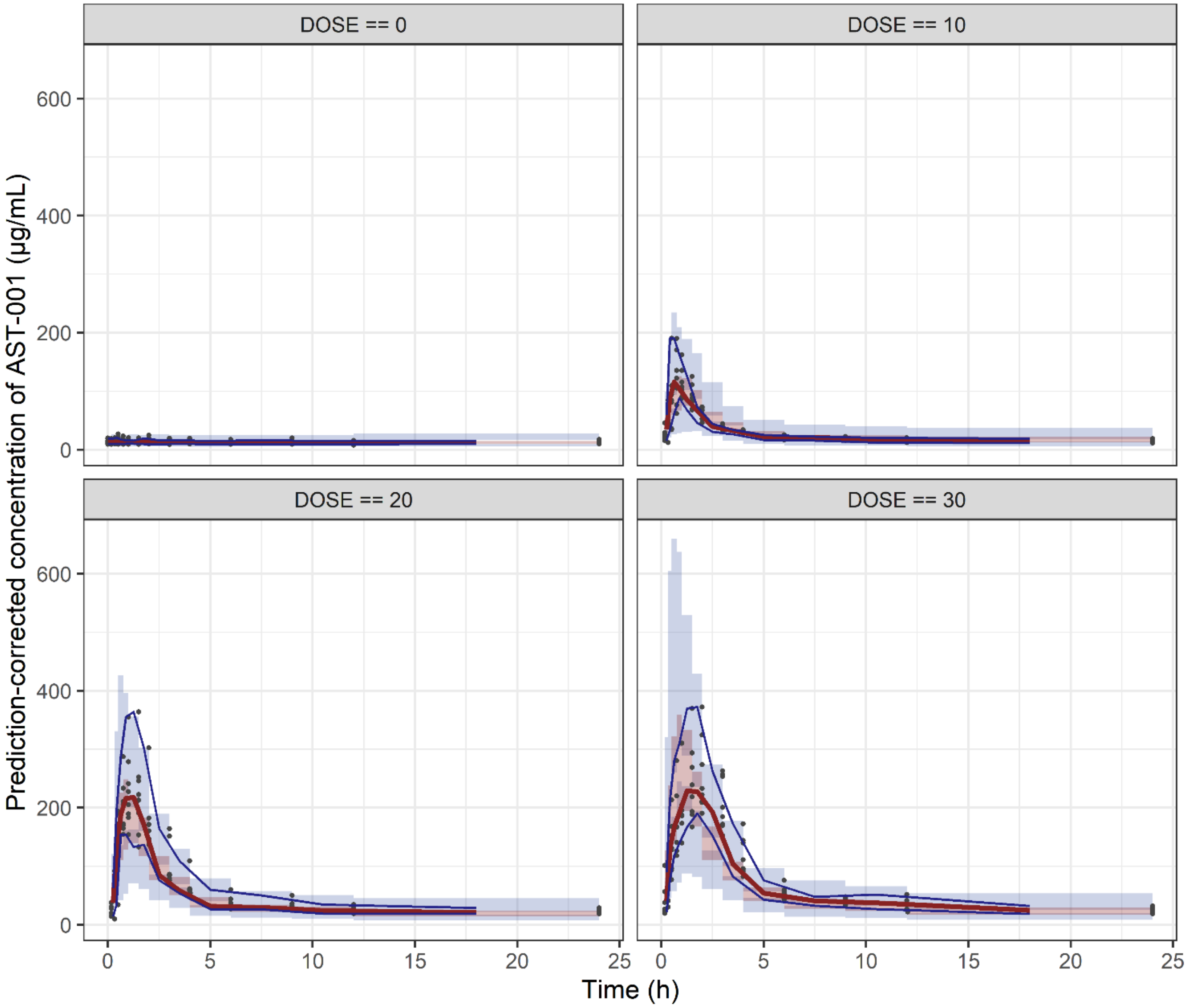

Supplementary Figure 3. Visual predictive check of final AST-001 model stratified by dose group. A total of 200 simulated dataset were generated. The closed circles represent the observed plasma AST-001 concentrations; the solid lines represent the 5th (blue), median (red), and 95th (blue) percentiles of the observed concentration; the blue and red areas indicate the 95% confidence interval of the simulated concentrations of each percentile.

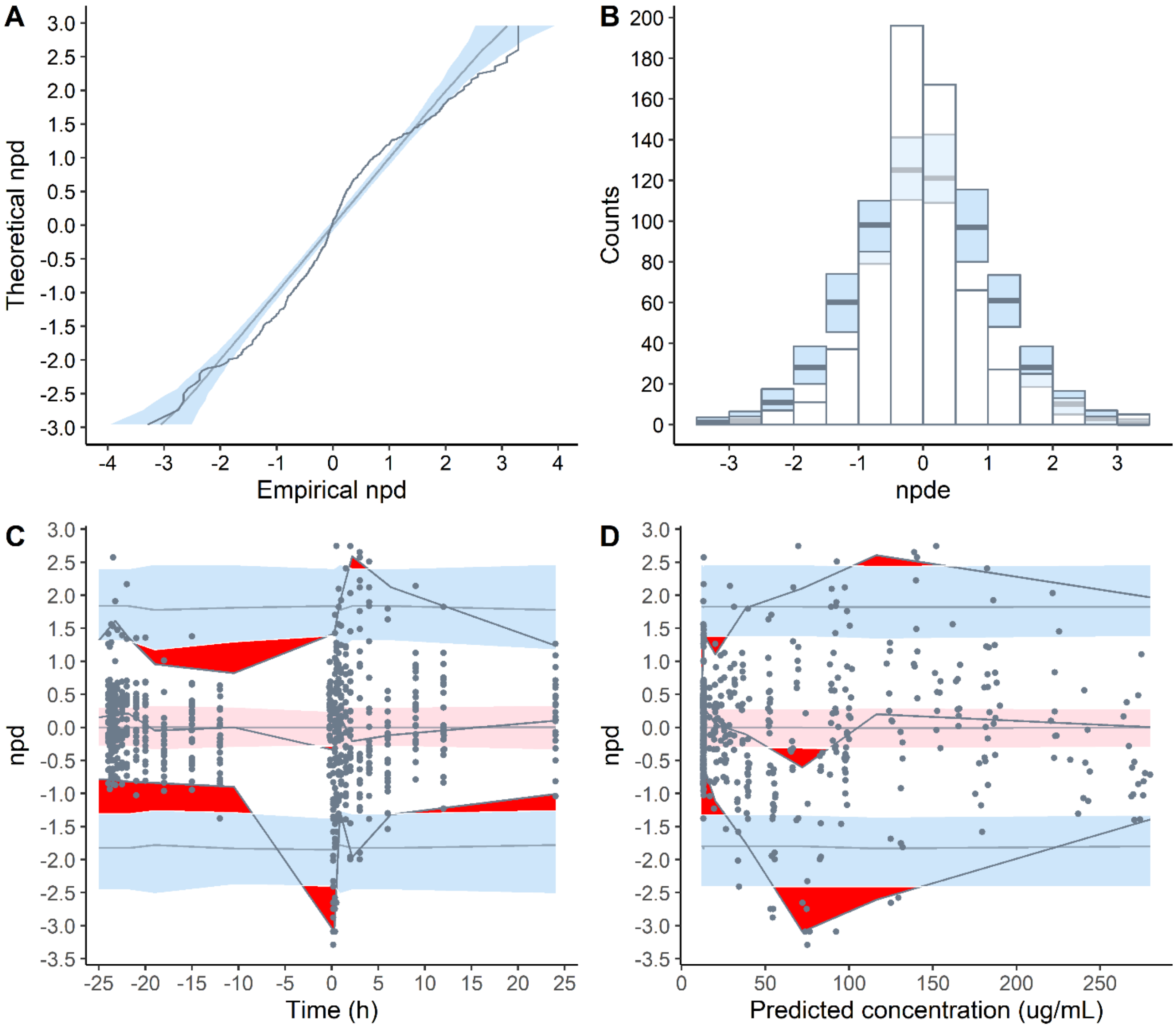

Supplementary Figure 4. Normalized Predictive Distribution Error (NPDE) of the final population PK model of AST-001. (A) Quantile-quantile plot of the NPDE, (B) histogram of the NPDE, (C) NPDE versus time, (D) NPDE versus individual predicted concentration.

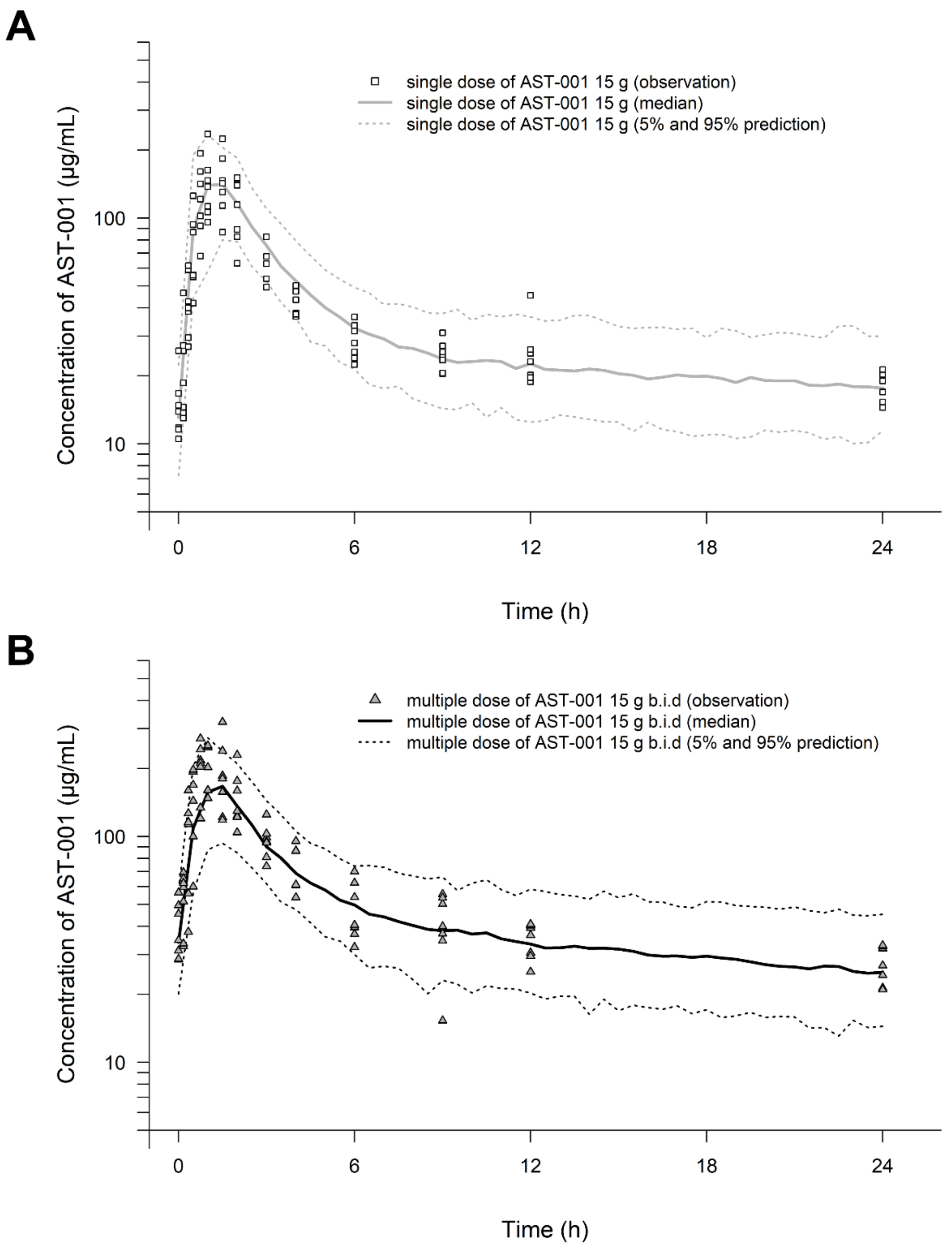

Supplementary Figure 5. Simulation results for the time-concentration profile of AST-001 after (A) single and (B) 7-day twice-daily multiple administration of 15 g dose of AST-001 overlaid with individual observations in healthy subjects. Line and dotted line indicate median, 5% and 95 % prediction intervals, respectively.

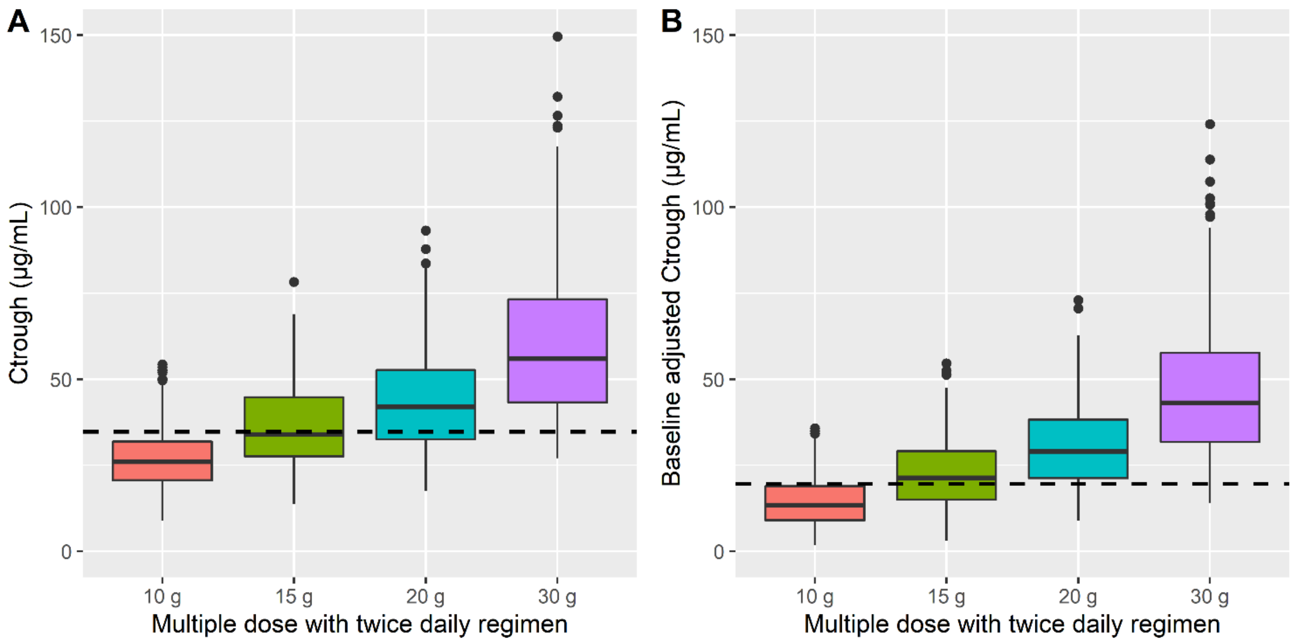

Supplementary Figure 6. Simulation results for (A) C_{trough} and (B) baseline-adjusted C_{trough} after 7-day twice daily multiple administration of 10, 15, 20, and 30 g dose of AST-001 in healthy subjects. Dotted line represents the observed median value after 7-day twice-daily multiple administration of 15 g dose of AST-001. C_{trough}, trough concentration.

1.2 Supplementary Tables

Supplementary Table 1. Summary of model development process for basic structure and inter-individual variability model and exploration of covariate model.

Phase	Model	Description	OFV
Base model development	1	1-compartmental model, first-order absorption, linear elimination	3637.441
	2	1-compartmental model, first-order absorption with lag time, linear elimination	3517.492
	3	1-compartmental model, first-order absorption, Michaelis-Menten elimination	3637.5
	4	1-compartmental model, first-order absorption with lag time, Michaelis-Menten elimination	3628.398
	5	2-compartmental model, first-order absorption, linear elimination	3441.869
	6	2-compartmental model, first-order absorption with lag time, linear elimination	3764.39
	7	2-compartmental model, first-order absorption, Michaelis-Menten elimination	3424.109
	8	1-compartmental model, zero-order absorption, linear elimination	3567.753
	9	1-compartmental model, zero-order absorption with lag time, linear elimination	4718.605
	10	1-compartmental model, zero-order absorption, Michaelis-Menten elimination	3567.646
	11	1-compartmental model, zero-order absorption with lag time, Michaelis-Menten elimination	4674.555
	12[a]	2-compartmental model, zero-order absorption, linear elimination	3401.16
IIV model development	13	2-compartmental model, zero-order absorption, linear elimination<br>IIV model: R1	3292.491
	14	2-compartmental model, zero-order absorption, linear elimination<br>IIV model: R1, CL	3251.877
	15	2-compartmental model, zero-order absorption, linear elimination<br>IIV model: R1, CL, V1	3046.441
	16	2-compartmental model, zero-order absorption, linear elimination<br>IIV model: R1, CL, V1, D1	2991.054

Phase	Model	Description	OFV
	**17**	**2-compartmental model, zero-order absorption, linear elimination**<br>**IIV model: R1, CL, V1, D1, correlation between CL & V1**	**2978.889**
Covariate model exploration	18[b]	2-compartmental model, zero-order absorption, linear elimination<br>IIV model: R1, CL, V1, D1, correlation between CL & V1<br>COV model: BW effect on CL	2976.679
	19[c]	2-compartmental model, zero-order absorption, linear elimination<br>IIV model: R1, CL, V1, D1, correlation between CL & V1<br>COV model: BW effect on CL and V1	2962.031
	20[d]	2-compartmental model, zero-order absorption, linear elimination<br>IIV model: R1, CL, V1, D1, correlation between CL & V1<br>COV model: BW effect on CL and Q	2966.482
	21[e]	2-compartmental model, zero-order absorption, linear elimination<br>IIV model: R1, CL, V1, D1, correlation between CL & V1<br>COV model: BW effect on CL, V1 and Q	2956.302
	22[f]	2-compartmental model, zero-order absorption, linear elimination<br>IIV model: R1, CL, V1, D1, correlation between CL & V1<br>COV model: BW effect on CL, V1, Q and V2	2952.459
	23	2-compartmental model, zero-order absorption, linear elimination<br>IIV model: R1, CL, V1, D1, correlation between CL & V1<br>Fixed allometric scaler of 0.75 for CL and 1 for V1	2967.306
	24	2-compartmental model, zero-order absorption, linear elimination<br>IIV model: R1, CL, V1, D1, correlation between CL & V1<br>Fixed allometric scaler of 0.75 for CL and Q, and 1 for V1 and V2	2958.579

[a] Selected as base structural model.
[b] Adding covariate model did not significantly improve the model (ΔOFV = -2.21).
[c] The estimated exponential coefficient for CL and V1 is 1.23 and 1.87, respectively, which is not feasible with the empirical allometric value of 0.75 and 1.
[d] The estimated exponential coefficient for CL and Q is 0.624 and 1.24, respectively, which is not feasible with the empirical allometric value of 0.75.

[e] The estimated exponential coefficient for CL, V1, and Q is 1.16, 1.58 and 1.04, respectively, which is not feasible with the empirical allometric value of 0.75 and 1.
[f] Minimization terminated.

Supplementary Table 2. Median, 5% and 95% percentile of simulation results of baseline-adjusted pharmacokinetic parameters after single and 7-day twice-daily multiple administration of 10, 15, 20, and 30 g doses of AST-001 in healthy subjects.

	**10 g**	**15 g**	**20 g**	**30 g**
**After single-dose administration**				
C_{max} (μg/mL)	95.8 [60.9-147.6]	152.4 [95-243.8]	189.5 [127.5-321.7]	302.8 [201.7-487.4]
AUC_{12h} (h*μg/mL)	288.7 [222.6-397.8]	457.6 [350.6-600.5]	602.1 [460.4-805.9]	924.4 [688.1-1232.9]
**After twice-daily multiple-dose administration**				
$C_{max,ss}$ (μg/mL)	111.3 [77.9-176]	179.2 [109.5-265.7]	229.5 [140.8-379.5]	336.5 [224.8-527]
AUC_{τ} (h*μg/mL)	417.5 [293.9-601]	638.7 [466.8-935]	873.6 [615.6-1236.7]	1300.8 [907.4-1940]

Values are presented as median [5th percentile - 95th percentile]

Supplementary Table 3. Summary of simulated pharmacokinetic parameters of AST-001 following fixed-dose twice-daily administration in the pediatric weight range.

Treatment group	$C_{max,ss}$ (µg/mL)					AUC_{τ} (h*µg/mL)				
	5%	25%	50%	75%	95%	5%	25%	50%	75%	95%
2g BID (10-14 kg)	98.7	131.1	158.6	190.2	265.5	566.7	751.9	891.8	1073.4	1431.6
4g BID (15-24 kg)	108.7	144.7	177.9	213.3	278.6	545.9	708.6	847.2	1006.2	1310.4
7g BID (25-37 kg)	120.7	155.8	187.1	229.8	303.9	555.8	709.7	838.9	982.0	1258.6
10g BID (38-51 kg)	123.0	159.2	188.7	233.3	301.4	571.3	706.5	826.8	958.6	1219.6
14g BID (52-60 kg)	136.8	174.0	211.8	251.1	324.7	611.1	759.1	888.0	1045.4	1300.3

$C_{max,ss}$, maximum plasma concentration at the steady state; AUC_{τ}, area under the curve over the dosing interval; BID, twice-daily regimen.
